# Clinical–radiological profile and risk factors for early mortality in preterm neonates with respiratory distress syndrome in Hoima, Western Uganda

**DOI:** 10.1038/s41598-025-31626-6

**Published:** 2025-12-13

**Authors:** Abdirahman Hussein Addow, Mohamed Jayte, Geoffrey Ofumbi Oburu, Simon Odoch, Jolly Kaharuza, Bappah Alkali, Yasin Ahmed H. Abshir, Hasssan Omar Ali, Walyeldin Elfakey

**Affiliations:** 1 Department of Paedtrics and Child Health, Faculty of Clinical Medicine and Dentistry, Kampala International University, Ishaka, Bushenyi, Kampala, Uganda; 2 Department of Internal Medicine, Faculty of Clinical Medicine and Dentistry, Kampala International University, Ishaka, Bushenyi, Kampala, Uganda

**Keywords:** Respiratory distress syndrome, Preterm neonates, Early mortality, Risk factors, Uganda, Diseases, Health care, Medical research, Risk factors

## Abstract

Respiratory distress syndrome (RDS) is the leading cause of respiratory failure and neonatal mortality, particularly in preterm infants. Despite global advances in neonatal care, RDS remains a significant problem in low-resource settings such as Uganda, where limited evidence exists on clinical profiles, mortality, and associated risk factors. Although these advances have greatly reduced mortality in high-income settings, their limited availability in Uganda contributes to the continued high burden of RDS-related deaths. To determine the clinical–radiological profile, early mortality, and risk factors for mortality among preterm neonates admitted with RDS at Hoima Regional Referral Hospital. A prospective cohort study was conducted among 150 preterm neonates with clinically and radiologically confirmed RDS. Data on sociodemographic, clinical, and obstetric characteristics were collected using structured questionnaires and chest X-rays. Participants were followed for seven days to determine outcomes. Descriptive statistics summarized baseline characteristics, while Poisson regression identified independent predictors of mortality. Of the 150 neonates, 62.7% were male and 70.7% were born before 32 weeks of gestation. Tachypnea (84.7%) and intercostal/subcostal retractions (71.3%) were the most frequent clinical features, while ground-glass patterns were the predominant radiological finding. Twenty-nine neonates died within the first seven days, giving an early mortality rate of 19.3%. Independent predictors of mortality were delayed presentation beyond six hours of life (aRR = 1.72, 95% CI: 1.43–2.07, *p* < 0.001) and birth weight < 1.5 kg (aRR = 1.12, 95% CI: 1.02–1.22, *p* = 0.015). RDS contributes substantially to early neonatal mortality in Uganda. Prompt recognition, early referral, and improved neonatal care—particularly for very low birth weight infants—are critical to improving outcomes. Although not directly measured in this study, improving access to antenatal corticosteroids and respiratory support—well-established interventions—remains essential for broader improvement of RDS outcomes.

## Introduction

Respiratory distress syndrome (RDS), also known as hyaline membrane disease, is the most common respiratory condition among premature newborns and a leading cause of neonatal mortality worldwide^1^. It is primarily caused by insufficient pulmonary surfactant production, leading to alveolar collapse, impaired gas exchange, hypoxemia, and respiratory failure^2^. Clinical manifestations include tachypnea, nasal flaring, grunting, intercostal retractions, and cyanosis, while radiological findings typically reveal diffuse atelectasis, low lung volumes, and a ground-glass appearance^3^. Despite major advances in neonatal intensive care, RDS continues to contribute substantially to morbidity and mortality, particularly in low- and middle-income countries (LMICs)^4^.

Globally, neonatal mortality remains high, with almost 40% of under-five deaths occurring in the neonatal period^5^. An estimated 3.9 million of the 10.8 million childhood deaths annually occur within the first 28 days of life, with RDS being a significant contributor^6^. In high-income countries, the sequential introduction of oxygen therapy, continuous positive airway pressure, surfactant therapy, and advanced ventilation techniques has dramatically reduced RDS-related mortality^7^. In contrast, LMICs continue to face higher mortality rates due to limited access to these interventions and shortages of trained personnel^8^. A recent meta-analysis reported pooled pediatric acute respiratory distress syndrome mortality at approximately 24%, with regional variation highlighting the disproportionate burden in low-resource settings^9^.

Sub-Saharan Africa carries nearly half (43%) of the world’s neonatal deaths^10^. In this region, RDS is consistently reported as one of the leading causes of respiratory failure and neonatal mortality^11^. In many African settings, preterm neonates often experience delays in accessing specialized neonatal care due to challenges with referral pathways, transportation, and limited stabilization at peripheral facilities, leading to late presentation to tertiary neonatal units^12^. For instance, studies in Tanzania and Ethiopia have reported mortality rates exceeding 30% among preterm neonates with RDS, with risk factors including low birth weight, absence of antenatal steroids, and delayed presentation^13^.

Uganda continues to experience a high neonatal mortality rate of 27 per 1000 live births, a figure that has stagnated over the last two decades despite improvements in child health^14^. Neonatal deaths contribute to 42% of all under-five mortality, with one in every sixteen children dying before their fifth birthday^15^. Respiratory distress syndrome is a major cause of these deaths, particularly among preterm neonates. However, there is limited local data on the clinical-radiological profile, mortality rates, and risk factors associated with early mortality in neonates with RDS^16^. Most available literature originates from outside Uganda, highlighting the urgent need for context-specific evidence to inform neonatal care practices^17^.

Respiratory distress syndrome affects up to 29% of preterm neonates admitted to neonatal intensive care units (NICUs), with mortality up to three times higher in East Africa compared to global averages^18^. Despite its high prevalence and burden, Uganda lacks studies describing the clinical features, radiological profile, and predictors of mortality among neonates with RDS^19^. Without such data, efforts to reduce neonatal mortality in line with the Sustainable Development Goal (SDG) target of reducing neonatal deaths to 12 per 1000 live births by 2030 remain hindered^20^. Understanding the burden and associated factors of RDS in the Ugandan context is therefore critical.

This study was conducted to address the knowledge gap regarding RDS among preterm neonates in Uganda. By characterizing the clinical-radiological profile, quantifying early mortality, and identifying risk factors for death, the findings will provide crucial evidence to guide neonatal care. The study will also contribute to local and global literature on neonatal RDS, help inform policy, improve resource allocation, and ultimately contribute to reducing neonatal mortality in Uganda^21^.

## Methods

### Study design and setting

This was a **hospital-based prospective cohort study** conducted at **Hoima Regional Referral Hospital (HRRH)**, located in Hoima City, western Uganda. HRRH serves as a referral hospital for eight surrounding districts and has a neonatal intensive care unit (NICU) where preterm neonates suspected of respiratory distress syndrome (RDS) are admitted and managed. The study was carried out in the Department of Paediatrics and Child Health.

HRRH neonatal unit is a Level II/III newborn care unit equipped with oxygen therapy, pulse oximetry, bubble CPAP, and phototherapy. Surfactant therapy and mechanical ventilation were not available during the study period. Antenatal corticosteroids are recommended in routine obstetric practice, although coverage remains inconsistent.

Chest radiographs were interpreted by both a paediatrician trained in neonatal respiratory disorders and a hospital radiologist. Imaging was performed within the first 6–24 h following admission.

Infants presenting after six hours of life were predominantly out-born, with delays attributed to transportation challenges and late referral from lower-level facilities. A minority of in-born infants experienced delayed admission due to prolonged resuscitation or stabilization in the labour ward. Hoima Regional Referral Hospital has a Level II neonatal unit with oxygen therapy and bubble CPAP. Mechanical ventilation and surfactant therapy were not available during the study period, and none of the infants with RDS received these interventions.

### Study population

The study population consisted of **all preterm neonates admitted with RDS** during the study period. The target population was all preterm neonates with RDS in the HRRH catchment area, while the accessible population was those presenting to HRRH during the study. Only neonates whose parents or guardians provided informed consent were enrolled.

### Eligibility criteria

Preterm neonates with clinical and radiological features of acute RDS were included. Those with features suggestive of RDS but later confirmed to have another diagnosis were excluded.

### Sample size and sampling

The sample size was determined using Daniel’s formula and data from previous studies. A total of **150 participants** were recruited, accounting for potential loss to follow-up. Consecutive sampling was used, enrolling all eligible neonates admitted with RDS until the sample size was achieved.

### Data collection procedures

Data were collected using a **structured**,** pretested questionnaire** capturing sociodemographic, obstetric, and clinical information. RDS diagnosis followed WHO and American Academy of Pediatrics criteria requiring at least two clinical signs plus characteristic radiographic findings. Infection in pregnancy’ included laboratory-confirmed infections (e.g., UTI), clinical diagnosis of chorioamnionitis, maternal intrapartum fever ≥ 38 °C, or prolonged rupture of membranes greater than 18 h^22^. Diagnosis required at least two clinical signs—such as tachypnea, grunting, nasal flaring, retractions, or cyanosis—plus one radiological finding. Anthropometric measurements, particularly birth weight, were taken using digital scales. Each neonate was followed for **seven days** to determine outcome (survival or death). Clinical assessments were conducted at least twice daily by the neonatal team during the seven-day follow-up. Although detailed clinical features were recorded, the study did not prospectively collect all parameters required to compute formal RDS severity scores such as Silverman–Anderson or Downes scores. Therefore, RDS severity classification was not performed.

### Quality assurance

The questionnaire was reviewed by pediatric experts to ensure content validity (Content Validity Index ≥ 0.80) and was pretested using the test–retest method on 10 neonates to ensure reliability (≥ 75%). Data collectors were trained, and daily checks were conducted by the principal investigator to ensure completeness and accuracy.

### Data management and analysis

Data were entered into **Excel** and exported to **SPSS version 26** for analysis. Descriptive statistics summarized baseline characteristics, and early mortality was calculated as the proportion of neonates who died within seven days. **Poisson regression** was used to identify independent risk factors, with results presented as adjusted rate ratios (aRR) and 95% confidence intervals. Statistical significance was set at *p* < 0.05. Variables with *p* < 0.20 in bivariate analysis were entered into the multivariable Poisson regression model.

## Result presentation

### Baseline characteristics of the study participants

In this study, we enrolled 150 preterm neonates with respiratory distress syndrome. Majority of these (80.7%), presented with in their first 6 h of life. Close to two thirds (62.7%) were male. Majority (70.7%) were born before 32 weeks of amenorrhea. The details of the baseline characteristics are shown in Table 1.

**Table 1:** Baseline characteristics of study participants.

Characteristic	Frequency	Percentage
**Age at presentation (hrs)**		
≤ 6 h	121	80.7
> 6 h	29	19.3
**Sex**		
Male	94	62.7
Female	56	37.3
**DM in pregnancy**		
No	128	85.3
Yes	22	14.7
**Infection in pregnancy**		
No	90	60.0
Yes	60	40.0
**Prenatal steroids**		
No	73	48.7
Yes	77	51.3
**Fever post delivery**		
No	117	78.0
Yes	33	22.0
**Gestational age**		
< 32	106	70.7
32+	44	29.3
**Birth weight**		
< 1.5	111	74.0
1.5+	39	26.0
**APGAR**		
< 7	26	17.3
7+	124	82.7
**Delivery mode**		
SVD	115	76.7
CS	35	23.3

SVD = spontaneous vaginal delivery, CS = Cesarean section. APGAR score refers to the 5-minute APGAR.

### Clinical-radiological profile of preterm neonates with respiratory distress syndrome (RDS) admitted at HRRH

**Fig. 1:**
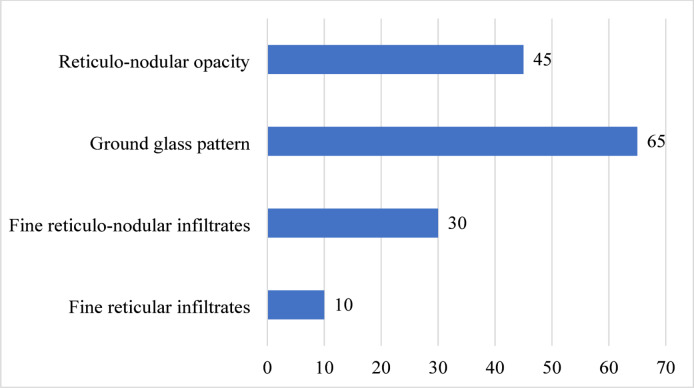
Radiological profile of preterm neonates with respiratory distress syndrome (RDS) admitted at HRRH.

Of the 150 participants enrolled with respiratory distress syndrome, the commonest clinical features were tachypnea and intercostal/subcostal recession seen in 84.7% and 71.3% of the participants respectively. The details of the clinical characteristics are shown in Table 2. The most common radiological findings were ground glass pattern (65/150), followed by reticulo-nodular opacity (45/150), fine reticulo-nodular infiltrates (30/150), and lastly fine reticular infiltrates seen in 10/150. None of the infants received surfactant or mechanical ventilation, and RDS care at Hoima consisted mainly of oxygen therapy and occasional bubble CPAP. This limited level of care may have influenced outcomes and affects generalizability. The details of the radiology patterns are shown in Fig. 1.

**Table 2:** Clinical profile of preterm neonates with respiratory distress syndrome (RDS) admitted at HRRH.

Clinical feature	Frequency	Percentage
**Tachypnea**		
No	23	15.3
Yes	127	84.7
**Recessions (intercostal/subcostal)**		
No	43	28.7
Yes	107	71.3
**Grunting**		
No	74	49.3
Yes	76	50.7
**Nasal flaring**		
No	82	54.7
Yes	68	45.3
**Cyanosis**		
No	136	90.7
Yes	14	9.3

### Early mortality among preterm neonates with RDS admitted at HRRH

**Fig. 2:**
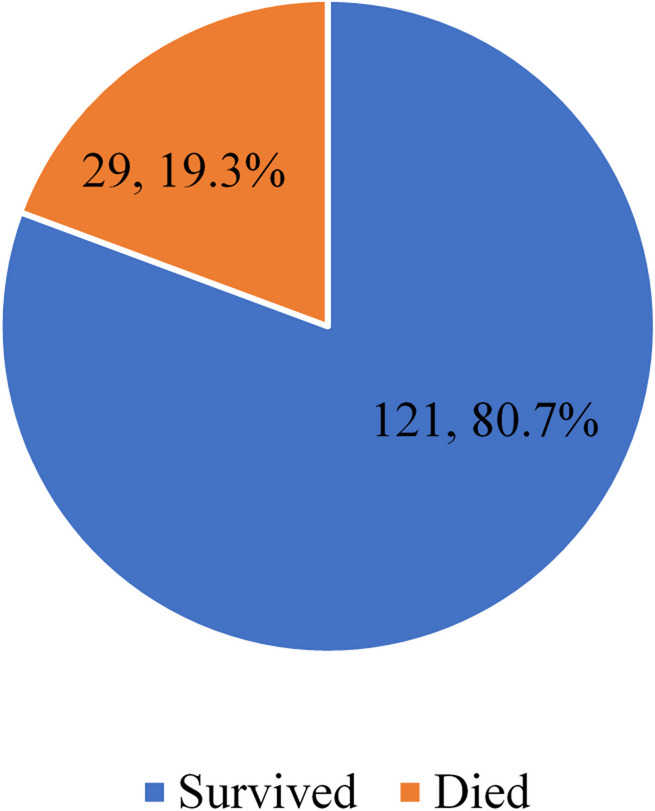
Early mortality among preterm neonates with RDS admitted at HRRH.

Of the 150 participants enrolled in the study, 29 passed away with in the first 7 days of life, representing a mortality rate of 19.3%, with a 95% confidence interval of 12.7–26.0%. The details are shown in Fig. 2.

### Risk factors for early mortality among preterm neonates with RDS admitted at HRRH

The variables considered for multivariate analysis (*P* < 0.2) were: age at presentation, prenatal use of steroids, birth weight, mode of delivery and presence of cyanosis. The details of bivariate analysis are shown in Table 3.

**Table 3:** Bivariable analysis of risk factors for early mortality among preterm neonates with RDS admitted at HRRH.

Characteristic	Survived, *N* = 121	Died, *N* = 29	Bivariable analysis		
			cRR	95% CI	*P* value
**Age at admission (hrs)**					
≤ 6 h	111(91.7)	10(34.5)	Ref		
> 6 h	10(8.3)	19(65.5)	1.773	1.481–2.122	**< 0.001**
**Sex**					
Male	73(60.3)	21(72.4)	1.084	0.957–1.228	0.205
Female	48(39.7)	8(27.6)	Ref		
**DM in pregnancy**					
No	101(83.5)	27(93.1)	Ref		
Yes	20(16.5)	2(6.9)	0.887	0.772–1.020	0.291
**Infection in pregnancy**					
No	71(58.7)	19(65.5)	Ref		
Yes	50(41.3)	10(34.5)	0.957	0.843–1.086	0.491
**Prenatal steroids**					
No	55(45.5)	18(62.1)	1.109	0.978–1.258	0.107
Yes	66(54.5)	11(37.9)	Ref		
**Fever post delivery**					
No	94(77.7)	23(79.3)	Ref		
Yes	27(22.3)	6(20.7)	0.985	0.848–1.145	0.847
**Gestational age**					
< 32	86(71.1)	20(69.0)	0.984	0.855–1.133	0.825
32+	35(28.9)	9(31.0)	Ref		
**Birth weight**					
< 1.5	83(68.6)	28(96.6)	1.254	1.141–1.379	**< 0.001**
1.5+	38(31.4)	1(3.4)	Ref		
**APGAR**					
< 7	21(17.4)	5(17.2)	0.999	0.845–1.180	0.988
7+	100(82.6)	24(82.8)	Ref		
**Delivery mode**					
SVD	90(74.4)	25(86.2)	Ref		
CS	31(25.6)	4(13.8)	0.902	0.792–1.027	0.119
**Tachypnea**					
No	19(15.7)	4(13.8)	Ref		
Yes	102(84.3)	25(86.2)	1.023	0.864–1.212	0.791
**Recessions (intercostal/subcostal)**					
No	34(28.1)	9(31.0)	Ref		
Yes	87(71.9)	20(69.0)	0.978	0.848–1.127	0.758
**Grunting**					
No	55(45.5)	19(65.5)	Ref		
Yes	66(54.5)	10(34.5)	0.882	0.778-1.000	0.250
**Nasal flaring**					
No	63(52.1)	19(65.5)	Ref		
Yes	58(47.9)	10(34.5)	0.919	0.812–1.040	0.282
**Cyanosis**					
No	112(92.6)	24(82.8)	Ref		
Yes	9(7.4)	5(17.2)	1.198	0.925–1.552	0.172
**Radiology patterns**					
Ground glass pattern	55(45.5)	10(34.5)	Ref		
Reticulo-nodular opacity	35(28.9)	10(34.5)	1.071	0.922–1.244	0.371
Fine reticulo-nodular	22(18.2)	8(27.6)	1.119	0.934–1.341	0.222
Fine reticular	9(7.4)	1(3.4)	0.948	0.771–1.164	0.608

cRR = Crude rate ratio, CI = Confidence interval, SVD = spontaneous vaginal delivery, CS = Cesarean section.

In the multivariable analysis, the independent risk factors for early mortality among preterm neonates with RDS were: presentation after six hours of life (aRR = 1.722, CI = 1.433–2.070, *P* < 0.001) and having a birth weight less than 1.5 kg (aRR = 1.118, CI = 1.022–1.223, *P* = 0.015). The mortality rate was increased by 72.2% among neonates who presented after six hours compared to those who presented in the first 6 h of life. The mortality rate was increased by 11.8% among neonates who were less than 1.5 kg compared to those who weighed ≥ 1.5 kg at birth. The details are shown in Table 4.

**Table 4:** Multivariable analysis of risk factors for early mortality among preterm neonates with RDS admitted at HRRH.

Characteristic	Bivariable analysis			Multivariable analysis		
	cRR	95% CI	*P* value	aRR	95% CI	*P* value
**Age at presentation (hrs)**						
≤ 6 h	Ref					
**> 6 h**	**1.773**	**1.481–2.122**	**< 0.001**	**1.722**	**1.433–2.070**	**< 0.001**
**Prenatal steroids**						
No	1.109	0.978–1.258	0.107	1.044	0.945–1.153	0.399
Yes	Ref					
**Birth weight**						
**< 1.5**	**1.254**	**1.141–1.379**	**< 0.001**	**1.118**	**1.022–1.223**	**0.015**
1.5+	Ref					
**Delivery mode**						
SVD	Ref					
CS	0.902	0.792–1.027	0.119	0.911	0.820–1.011	0.178
**Cyanosis**						
No	Ref					
Yes	1.198	0.925–1.552	0.172	1.028	0.812–1.302	0.816

cRR = Crude rate ratio, aRR = adjusted rate ratio, CI = Confidence interval.

.

## Discussion

This study investigated the clinical–radiological profile and risk factors for early mortality among preterm neonates with respiratory distress syndrome (RDS) admitted at Hoima Regional Referral Hospital. The findings show that tachypnea and intercostal/subcostal recession were the most common clinical features, while ground-glass patterns and reticulo-nodular opacities predominated on chest radiographs. These findings are consistent with studies in Iran and Italy that reported tachypnea, retractions, and grunting as common clinical presentations in neonates with RDS^23,24^. Similarly, radiological profiles such as reticulonodular opacities and ground-glass appearances have been described as pathognomonic features of neonatal RDS^25^.

The study found a **19.3% early mortality rate**, which aligns with pooled estimates of pediatric acute respiratory distress syndrome mortality at 18–24%^9,26^. Our mortality rate (19.3%) is lower than that reported in Tanzania (31.3%) but comparable to that in China (18.2%)^27^. The observed mortality in Uganda underscores the persistent gap in neonatal survival outcomes compared to high-income countries, where advances such as surfactant therapy and continuous positive airway pressure have significantly reduced deaths. In high-income settings, early mortality (within the first 7 days) from respiratory distress syndrome is markedly lower, typically ranging from 3 to 5% in the United States, 4–6% in the United Kingdom, and below 5% in most Western European neonatal intensive care units, compared to substantially higher rates reported in low-resource countries such as Uganda^7^.

Key independent risk factors for early mortality in this study were **presentation after six hours of life** (*p* < 0.001) and **birth weight below 1.5 kg** (*p* = 0.015), both of which remained statistically significant in the multivariable logistic regression analysis. These findings are in line with evidence from Tanzania and Ethiopia, where low birth weight, delayed presentation, and lack of antenatal steroid use were significant predictors of poor outcomes^13,27,28^. The biological plausibility is clear: very low birth weight neonates have immature lungs with insufficient surfactant production, increasing their susceptibility to alveolar collapse and hypoxemia^2^. Delayed presentation may lead to progression of hypoxia and irreversible organ damage before effective interventions are initiated.

The persistence of high RDS-related mortality in Uganda reflects systemic challenges, including limited access to surfactant, advanced respiratory support, and neonatal intensive care^29,30^. This highlights the urgent need for **early recognition**,** timely referral**,** and appropriate resource allocation** to neonatal services. Strengthening antenatal care, particularly maternal corticosteroid administration, and ensuring availability of neonatal resuscitation and respiratory support technologies are critical to reducing mortality.

Strengthening neonatal care services is essential to reduce RDS-related mortality in Uganda. Early identification and prompt referral of preterm neonates with respiratory distress should be prioritized, with emphasis on **timely presentation within the first six hours of life**. Availability and accessibility of surfactant therapy, continuous positive airway pressure, and skilled neonatal intensive care must be expanded. In addition, scaling up **antenatal corticosteroid use**, improving obstetric care, and routine training of healthcare workers in neonatal resuscitation are critical. Policymakers should allocate adequate resources to neonatal units, while future research should focus on context-specific interventions to improve outcomes for very low birth weight infants.

### Strengths and limitations

This study is among the first in Uganda to comprehensively describe the clinical–radiological profile and risk factors for early mortality in preterm neonates with RDS, providing locally relevant evidence to guide neonatal care. The **prospective cohort design** and systematic follow-up for seven days strengthened the reliability of outcome assessment, while the use of both clinical and radiological criteria improved diagnostic accuracy. However, the study was conducted in a single regional referral hospital, which may limit the generalizability of findings to other settings. Additionally, the relatively small sample size constrained the power to detect some associations, and resource limitations, such as restricted access to surfactant therapy and advanced ventilation, may have influenced outcomes.

## Conclusions

This study demonstrates that respiratory distress syndrome remains a major contributor to early neonatal mortality in Uganda, with nearly one in five preterm neonates dying within the first week of life. Tachypnea and intercostal retractions were the most frequent clinical findings, while ground-glass patterns and reticulo-nodular opacities dominated the radiological profile. Early mortality was strongly associated with delayed presentation beyond six hours of life and very low birth weight (< 1.5 kg). Although these advances have greatly reduced mortality in high-income settings, their limited availability in Uganda contributes to the continued high burden of RDS-related deaths. Strengthening neonatal services is essential if Uganda is to reduce RDS-related deaths and achieve the global target for neonatal survival set under the Sustainable Development Goals.

## Data Availability

The datasets generated and analyzed during the study are available from the corresponding author upon reasonable request.
